# p53 mutations in urinary bladder cancer

**DOI:** 10.1054/bjoc.2001.1823

**Published:** 2001-06

**Authors:** P Berggren, G Steineck, J Adolfsson, J Hansson, O Jansson, P Larsson, B Sandstedt, H Wijkström, K Hemminki

**Affiliations:** Department of Biosciences at Novum, Karolinska Institute, 141 57 Huddinge, Sweden; Clinical Cancer Epidemiology and Clinical Oncology, Department of Oncology-Pathology, Karolinska Hospital, 171 76 Stockholm, Sweden; Oncology Centre, Stockholm City Council, 171 76 Stockholm, Sweden; Department of Urology, Karolinska Hospital, 104 01 Stockholm, Sweden; Emergency Clinic, Karolinska Hospital, 171 76 Stockholm, Sweden; Department of Pathology, Karolinska Hospital, 104 01 Stockholm; Department of Urology, Huddinge University Hospital, 141 86 Huddinge, Sweden

**Keywords:** p53, urinary bladder cancer, PCR, F-SSCP

## Abstract

We have screened for mutations in exons 5–8 of the *p53* gene in a series consisting of 189 patients with urinary bladder neoplasms. 82 (44%) neoplasms were lowly malignant (Ta, G1–G2a) and 106 (56%) were highly malignant (G2b–G4 or ≥T1). Only one mutation was in a lowly malignant urinary bladder neoplasm, in total we found p53 mutations in 26 (14%) of the 189 patients. 30% of the samples had loss of heterozygosity (LOH) for one or both of the p53 exogenic (CA)n repeat and the p53 intragenic (AAAAT)n repeat markers. 31 samples (21%) showed LOH but were not mutated, suggesting other mechanisms inactivating p53 than mutations. 4 mutations were found at codon 280 and 2 mutations were found at codon 285, 2 previously reported hot spots for urinary bladder cancer. The study indicate a boundary between G2a and G2b tumours concerning the occurrence of genetic events affecting p53 function; moderately differentiated (G2) urinary bladder neoplasms probably are genetically heterogeneous which supports the suggestion that they should not be grouped together but instead, for example, be categorized as either lowly or highly malignant. © 2001 Cancer Research Campaign http://www.bjcancer.com


*British Journal of Cancer* (2001) **84**(11), 1505–1511
© 2001 Cancer Research Campaign
doi: 10.1054/ bjoc.2001.1823, available online at http://www.idealibrary.com on IDEAL®

### p53 mutations in urinary bladder cancer

**P Berggren[1,2], G Steineck[2], J Adolfsson[3], J Hansson[2], O Jansson[4], P Larsson[5], B Sandstedt[6], H Wijkström[7] and K Hemminki[1]**

[1]Department of Biosciences at Novum, Karolinska Institute, 141 57 Huddinge, Sweden; [2]Clinical Cancer Epidemiology and Clinical Oncology, Department of Oncology-Pathology, Karolinska Hospital, 171 76 Stockholm, Sweden; [3]Oncology Centre, Stockholm City Council, 171 76 Stockholm, Sweden; [4]Department of Urology, Karolinska Hospital, 104 01 Stockholm, Sweden; [5]Emergency Clinic, Karolinska Hospital, 171 76 Stockholm, Sweden; [6]Department of Pathology, Karolinska Hospital, 104 01 Stockholm; [7]Department of Urology, Huddinge University Hospital, 141 86 Huddinge, Sweden

**Summary** We have screened for mutations in exons 5–8 of the *p53* gene in a series consisting of 189 patients with urinary bladder neoplasms. 82 (44%) neoplasms were lowly malignant (Ta, G1–G2a) and 106 (56%) were highly malignant (G2b–G4 or ≥T1). Only one mutation was in a lowly malignant urinary bladder neoplasm, in total we found p53 mutations in 26 (14%) of the 189 patients. 30% of the samples had loss of heterozygosity (LOH) for one or both of the p53 exogenic (CA)n repeat and the p53 intragenic (AAAAT)n repeat markers. 31 samples (21%) showed LOH but were not mutated, suggesting other mechanisms inactivating p53 than mutations. 4 mutations were found at codon 280 and 2 mutations were found at codon 285, 2 previously reported hot spots for urinary bladder cancer. The study indicate a boundary between G2a and G2b tumours concerning the occurrence of genetic events affecting p53 function; moderately differentiated (G2) urinary bladder neoplasms probably are genetically heterogeneous which supports the suggestion that they should not be grouped together but instead, for example, be categorized as either lowly or highly malignant. © 2001 Cancer Research Campaign http://www.bjcancer.com

**Keywords**: p53; urinary bladder cancer; PCR; F-SSCP

The human tumour suppressor gene *p53* maps to chromosome 17p13.1, consists of 11 exons spanning over 20 kb of DNA and encodes for a 393 amino acids, 53kDa nuclear protein (Lane and Crawford, 1979; Isobe et al, 1986). The p53 protein has several biological functions such as involvement in cell cycle regulation, programmed cell death, senescence, differentiation and development, transcription, DNA replication, DNA repair and maintenance of genomic stability (Hainaut and Hollstein, 2000). Genetic changes in the *p53* gene are found in almost every kind of human cancer (Hainaut et al, 1998; Hainaut and Hollstein, 2000). Missense mutations can induce alterations in the tertiary structure of the protein, thereby interfering with the ability of p53 to bind to recognition sequences (Kern et al, 1991) and/or activate transcription (Fields and Jang, 1990; Raycroft et al, 1990). Mutations are diverse in localization and nature; even though mutations are found outside the evolutionary conserved regions, the majority of point mutations are located within these (exons 5–8) (Greenblatt et al, 1994; Hainaut and Hollstein, 2000).

Urinary bladder neoplasms are the forth most common neoplasm in men in the Western world (Knowles, 1999b). The incidence in Sweden is about 32 for men and 9 for women per $10^5$ person-years (approximately 1500 and 600 cases, respectively) (Centre for Epidemiology, 1998). Almost all bladder cancers in Western countries are transitional cell carcinomas (TCC). Cigarette smoking, industrially related aromatic amines and exposure to the drugs phenacetine, chlornaphrazine and cyclophosphamide, are connected to TCC occurrence (IARC, 1985; Steineck et al, 1995; Cohen et al, 2000). Underlying molecular defects involve the activation of oncogenes and inactivation of tumour suppressor genes and many genetic alterations have been identified, of which some of the affected genes are *p53*, *p16*, *p14^ARF^*, *PTEN*, *PTCH*, *DBCCR1* and *RB* (Knowles, 1999a). The histopathological classification has recently been the subject of an intense debate. After initial work by, for example, Bergqvist et al (1965) and Esposti and Zajicek (1972), some centres have modified the WHO grading system to distinguish grade 2a and grade 2b tumours, the outcome between these subgroups differ markedly in some series (Malmstrom et al, 1987; Malmstrom, 1988; Carbin et al, 1991). Recently an international group of pathologists suggested that moderately differentiated (G2) urinary bladder neoplasms should be either categorized as lowly or highly malignant (Epstein et al, 1998). The proposed division is similar, if not identical, to the previously described distinction between G2a and G2b.

Previous studies of p53 mutations in urinary bladder neoplasms have reported mutation frequencies between 6% and 61% (Shipman et al, 1997; Sidransky et al, 1991). The diverse frequencies are to some extent determined by variations in the tumour stage and grade: a much higher number of mutations are found in tumours of high stage and grade than in tumours of low stage and grade (Fujimoto et al, 1992). We have analysed the p53 mutational spectra for urinary bladder neoplasms by studying the mutation frequency in exons 5–8 of the *p53* gene in a material consisting of TCCs of various stage and grade. We used a highly sensitive multiplex-PCR and fluorescent SSCP method for mutation screening followed by sequencing for confirmation and identification of mutations. Analyses of the mutational spectra for the *p53*

*Received 16 October 2000*
*Revised 22 February 2001*
*Accepted 28 February 2001*

*Correspondence to:* P Berggren. E-mail: petra.berggren@cnt.kise gene may provide clues about cancer aetiology, mechanisms of mutations and the role that p53 inactivation plays in urinary bladder cancer.

### MATERIALS AND METHODS

Tumour tissues from almost all newly diagnosed cases of urinary bladder cancer during the years 1995 and 1996 were collected in the Stockholm area. We have more than 600 cases, which are almost all cases that occurred in the area at the time. Of the total number of urinary bladder neoplasms collected, 189 samples were selected from cases where corresponding normal tissue and aetiological information was available. None of the patients had been given prior treatment before analysis. All cases were screened for mutations and polymorphisms, using multiplex-PCR fluorescent single-strand conformation polymorphism (M-PCR-F-SSCP), between the regions coding for exons 5–8 of the *p53* gene. Base changes present in both normal and tumour tissues at a frequency higher than 1% were defined as polymorphisms; changes present in tumour tissue only were defined as mutations. Samples positive for mutations or polymorphisms were sequenced twice from separate PCRs to exclude artefacts.

### Patients and tissue

Tumours were removed with transurethral resection. 4 tissue samples were taken with cold cup biopsy and snap frozen in –80˚C before removal. Frozen tissues were cut in approximately 5 μm thick sections. The first and last sections were stained and examined for tumour contents by a pathologist. Only biopsies with more than 70% tumour cells were included in the present analysis. Tumour DNA was extracted by a previously described method (Sambrook et al, 1989). All tumours were staged and graded. Stage was assessed according to the TNM-system (Hall and Prout, 1990). All muscle invasive tumours were analysed together. Grading was done according to Bergkvist (Bergkvist et al, 1965). Stage and grade information was available for 188 of the 189 bladder neoplasms and distributed as follows: Tis: 3 (1.6%), Ta: 106 (56%), T1: 29 (15%) and ≥T2: 50 (27%). G1: 10 (5%), G2a: 78 (41%), G2b: 33 (18%), G3: 64 (34%) and G4: 3 (1.6%). Combined information gives 82 (44%) lowly malignant neoplasms (Ta, G1–G2a) and 106 (56%) high-grade (G2b–G4) or invasive (≥T1) highly malignant neoplasms. From each patient venous blood was drawn into EDTA tubes and frozen. DNA was extracted as previously described (Wada et al, 2000).

### Multiplex PCR and fluorescent SSCP

We used a mutation screening method developed in our laboratory, detailed PCR-SSCP conditions and primer sequences have been previously described (Berggren et al, 2000b). In short, exons 5–8 of the *p53* gene were amplified in a single PCR reaction and then loaded onto native SSCP gels using different conditions and an ABI 377 (PE Biosystems). Mutations and polymorphisms were detected as mobility shifts. All mobility shifts were sequenced with DyeDeoxy terminator cycle sequencing. Thermosequenase 2.0 (Amersham Pharmacia Biotech) or Big Dye (PE Biosystems) sequencing kits were used according to the manufacturer's instructions. Putative mutations were confirmed on both coding and noncoding strands from a new PCR reaction. The sample with a deletion (K105) was reamplified in a radioactive PCR and loaded onto a slab gel from which the band-shift was excised and sequenced.

### Detection of LOH

Cases in which both tumour and normal tissue were available (175 patients) were analysed for loss of heterozygosity (LOH). 2 markers were used: the p53 exogenic $(CA)_n$ dinucleotide and the intragenic (intron one) $(AAAAT)_n$ pentanucleotide repeat polymorphisms. Tumour-and normal DNA was amplified using previously reported primers and PCR conditions (Futreal et al, 1991; Jones and Nakamura, 1992). PCR fragments, labelled with Cy-5, were loaded onto a denaturing (9 M urea), 6% acrylamide gel, run for 80 minutes at 900 V, 40 mA, 20 W and 45˚C in 1 × TBE buffer using an ALF express machine (Amersham Pharmacia Biotech). Size markers of 100 and 150 base pairs were included in the gel. LOH was defined as a reproducible reduction of at least 30% of either the smaller or larger allele. Samples that were homozygous for the marker were defined as non-informative and excluded from calculations for LOH frequency. All samples where LOH was found were re-analysed from a new PCR.

### Statistical calculations

The correlations between mutation and LOH versus stage (Tis, Ta, T1 and ≥T2) and grade (G1, G2a, G2b, G3 and G4) were done with regression analysis using Microsoft Excel 97. The relationships between mutation and LOH versus lowly malignant tumours (Ta, G1–G2a) and highly malignant tumours (G2b–G4 and ≥T1) where calculated with a $\chi^2$ test using EPI6 (CDC, USA and WHO Geneva, Switzerland).

### RESULTS

In the 189 samples analysed, 31 mutations in 26 patients (14%) and 32 polymorphisms were found, all mutations are shown in Table 1. One of 82 patients (1.2%) with a lowly malignant tumour (Ta, G1–G2a) had a p53 mutation as did 25 of 106 (24%) with a high-grade (G2b–G4) or invasive (≥T1) tumour. Tumour stage and grade were associated to p53 mutation and LOH of both the $(CA)_n$ repeat and the $(AAAAT)_n$ repeat. Lowly malignant neoplasms had a lower percentage of LOH (12%, 12%) than highly malignant neoplasms (30%, 43%) for both the $(CA)_n$ repeat and the $(AAAAT)_n$ repeat.

### Mutations

22 of the mutations were transitions, 16 G:C → A:T and 6 A:T → G:C. 8 mutations were transversions, 6 G:C → C:G, one G:C → T:A and one A:T → T:A. No A:T → C:G mutations were found. 7 mutations were in exon 5, 5 in exon 6, 7 in exon 7 and 12 in exon 8. 3 were silent, 22 were missense mutations, 5 were nonsense mutations and one mutation was a deletion. The 6 base pairs deletion was found in exon 7, in the same allele as the deletion and right next to it, there was also a point mutation. 4 of the 5 nonsense mutations were found in exon 6. A silent mutation in codon 153 was present in both normal and tumour tissue and has been reported by others as a silent mutation found in tumour tissue only, we therefore believe that the one found by us is a germline mutation (Lohmann et al, 1993;

 © 2001Cancer Research Campaign

**Table 1** Mutations, loss of heterozygosity, stage and grade

| Sample | Exon | Codon | Change | Triplet | Amino acid | $(CA)_n$ | $(AAAAT)_n$ | Stage | Grade |
|---|---|---|---|---|---|---|---|---|---|
| K131 | 5 | 163* | A→G | TAC→TGC | Tyr→Cys | I | I | Ta | G2a |
| SS11 | 5 | 153** | C→T | CCC→CCT | Pro→Pro | I | I | Ta | G2b |
| K37 | 6 | 196 STOP | C→T | CGA→TGA | Arg→OPA | NI | NI | Ta | G2b |
| D36 | 6 | 192 STOP | C→T | CAG→TAG | Gln→AMB | NI | NI | Ta | G3 |
| K95 | 7 | 245 | G→C | GGC→CGC | Gly→Arg | NTNA | NTNA | Ta | G3 |
| H33 | 8 | 281 | G→C | GAC→CAC | Asp→His | LOH | LOH | Ta | G3 |
| S139 | 6 | 192 STOP* | C→T | CAG→TAG | Gln→AMB | NI | LOH | T1 | G3 |
| K130 | 6 | 213 STOP* | C→T | CGA→TGA | Arg→OPA | LOH | LOH | T1 | G3 |
| D35 | 7 | 236 | A→G | TAC→TGC | Tyr→Cys | LOH | NI | T1 | G3 |
| S77 | 7 | 239 | A→G | AAC→AGC | Asn→Ser | LOH | LOH | T1 | G3 |
| K105 | 7 | 247(14066) | A→G | AAC→GGG | Asn→Gly | NI | NI | T1 | G3 |
| | | 14067-72 | ACCGGA | -6bp | Deletion | | | | |
| K111 | 7 | 248 | C→T | CGG→TGG | Arg→Trp | I | I | T1 | G3 |
| | 8 | 280 | G→A | AGA→AAA | Arg→Lys | | | | |
| S111 | 7 | 248 | G→A | CGG→CAG | Arg→Gln | NI | LOH | T2 | G2a |
| K119 | 5 | 171* | G→C | GAG→CAG | Glu→Gln | NI | NI | T2 | G2b |
| | 5 | 175* | G→A | CGC→CAC | Arg→His | | | | |
| K94 | 8 | 280 | G→C | AGA→ACA | Arg→Thr | NTNA | NTNA | T2 | G2b |
| K62 | 5 | 163 | A→G | TAC→TGC | Tyr→Cys | LOH | LOH | T2 | G3 |
| K140 | 5 | 179* | A→G | CAT→CGT | His→Arg | NI | LOH | T2 | G3 |
| K48 | 5 | 179 | C→T | CAT→TAT | His→Tyr | LOH | NI | T2 | G3 |
| H48 | 6 | 197 | G→A | GTG→ATG | Val→Met | LOH | LOH | T2 | G3 |
| S174 | 8 | 273 | G→A | CGT→CAT | Arg→His | I | NI | T2 | G3 |
| S104 | 8 | 280 | G→A | AGA→AAA | Arg→Lys | NI | NI | T2 | G3 |
| S37 | 8 | 283 | G→C | CGC→CCC | Arg→Pro | NTNA | NTNA | T2 | G3 |
| H51 | 8 | 285 | A→T | GAG→GTG | Glu→Val | NTNA | NTNA | T2 | G3 |
| S27 | 8 | 286 | G→T | GAA→TAA | Glu→OCH | I | LOH | T2 | G3 |
| D79 | 8 | 280 | G→C | AGA→ACA | Arg→Thr | NI | NI | T2 | G4 |
| D60 | 8 | 285 | G→A | GAG→AAG | Glu→Lys | NI | LOH | T2 | G4 |
| | | 287 | G→A | GAG→GAA | Glu→Glu | | | | |
| | | 291 | G→A | AAG→AAA | Lys→Lys | | | | |

*The mutation has been previously reported (Berggren et al, 2000b), **Germline mutation. I = informative, NI = not informative, LOH = loss of heterozygosity, NTNA = normal tissue not available.

Bringuier et al, 1998). 6 mutations were at CpG sites. The mutations we found in codons 153, 171, 197, 239, 245, 247, 281, 286 and 291 have not been previously reported for urinary bladder cancer, though all the mutations, but the ones in codons 171 and 247, have been reported for other cancer types (Hainaut et al, 1998). 2 samples had double missense mutations, one sample with 2 mutations in exon 5 and one sample with a mutation in exon 7 and 8. One sample had 3 mutations, all 3 G:C → A:T transitions, in exon 8 in codons 285, 287 and 291. The codon 285 mutation was a missense mutation but the other 2 were silent. Representative SSCP results are shown in Figure 1.

### Polymorphism

32 polymorphisms were found. 3 of them were A:T → G:C transitions at codon 213 in exon 6 (Carbone et al, 1991), occurring at a frequency of 1.6%. Moreover 29 were linked C:G → T:A and T:A → G:C polymorphisms (24 of these have been previously reported (Berggren et al, 2000a)) in intron 7 at base positions 14181 and 14201 (Prosser and Condie, 1991) (genebank accession no. X54156) occurring at a frequency of 15%.

### LOH

14 of the tumour samples were excluded from LOH analysis because there was no normal tissue available. 2 samples analysed for the $(CA)_n$ marker failed. 58 (34%) of the 173 samples analysed for the $(CA)_n$ repeat were homozygous and thus not informative for the marker. Of the 115 (66%) informative samples 90 (78%) retained heterozygosity and 25 (22%) showed LOH. 72 of the 175 samples analysed for the $(AAAAT)_n$ repeat were homozygous. Of the 103 (59%) informative samples, 72 (70%) retained heterozygosity and 31 had LOH (30%). 43 (29%) of the samples informative for at least one of the repeats showed LOH for one or both repeats. Only one sample with LOH for the $(CA)_n$ repeat retained heterozygosity for the $(AAAAT)_n$ repeat whereas 8 samples that had LOH for the $(AAAAT)_n$ repeat retained heterozygosity for the $(CA)_n$ repeat.

### Mutation and LOH

For 4 of the mutated samples there was no normal tissue available. 7 of the mutated samples showed LOH for the $(CA)_n$ repeat, 10 were not informative and 5 samples retained heterozygosity. 10 of the mutated samples showed LOH for the $(AAAAT)_n$ repeat, 9 were not informative and 3 samples retained heterozygosity. One sample informative but without LOH for the $(CA)_n$ repeat showed LOH for the $(AAAAT)_n$ repeat. There were 3 mutated samples that were informative but without LOH for both repeats. One of these samples had double mutations (exon 7 and 8), one had a silent mutation (codon 153) and the third sample was the only mutated sample that was of both low grade and low stage. In 31 samples (21% of all samples informative for at least one of the repeats) showing LOH for either the $(CA)_n$ or the $(AAAAT)_n$ repeat, or both repeats, we could not find any mutations in any of the exons 5, 6, 7 or 8.

© 2001 Cancer Research Campaign

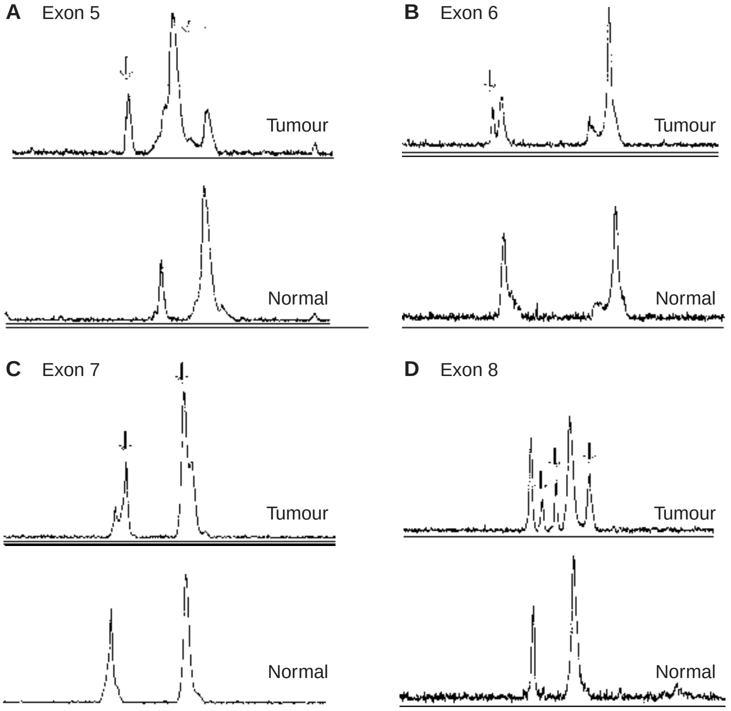

**Figure 1** Representative pictures from SSCP electropherograms for the 4 amplified fragments, p53 exons 5–8. Top panels show tumour tissues and lower panels show wild-type tissues. Arrows point at additional peaks. Panel **A** is sample K119, **B** is sample D36, **C** is sample K105 and **D** is sample D60. SSCP conditions for panels **A** and **B** were 0.6 × MDE with 5% glycerol run at 33˚C and conditions for panels **C** and **D** were 0.5 × MDE with 5% glycerol and 1 M urea run at 20˚C

### Stage and grade versus mutation

A higher proportion of mutations was found in tumours of high stage or grade than in tumours of low stage or grade. The correlation is shown in Figure 2, panels A and B. Tis: 0/3 (0%), Ta: 6/106 (6%), T1: 6/29 (21%) and ≥ T2: 14/50 (28%) ($P$ = 0.02). G1: 0/10 (0%), G2a: 2/78 (3%), G2b 4/33 (12%), G3: 18/64 (28%) and G4: 2/3 (67%) ($P$ = 0.03). Stage and grade information taken together show that lowly malignant tumours (Ta, G1–G2a) carry fewer mutations (1/82 = 1.2%) than highly malignant tumours (high grade (G2b–G4) or invasive (≥ T1)) where 25 of 106 (24%) had p53 mutations ($P$ = 0.00001).

### Stage and grade versus LOH

A higher number of tumours of high stage or grade had loss of heterozygosity for the $(CA)_n$ repeat than did tumours of low stage or grade. Tis: 0/2 (0%), Ta: 11/66 (17%), T1: 6/22 (27%), ≥T2: 8/25

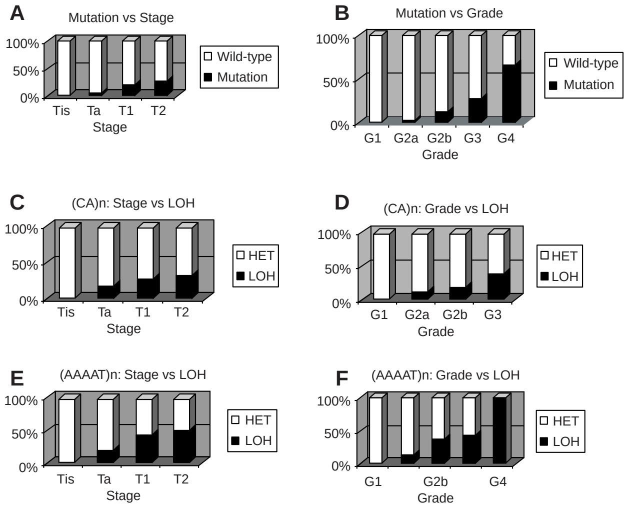

**Figure 2** Correlation between stage, grade, mutations and loss of heterozygosity. Panels **A** and **B** show mutations on y-axis and stage and grade on x-axis. Panels **C**–**D** show LOH for the $(CA)_n$ repeat on the y-axis and stage and grade on the x-axis. Panels **E**–**F** show LOH for the $(AAAAT)_n$ repeat on the y-axis and stage and grade on the x-axis

 *© 2001Cancer Research Campaign*

(32%) ($P$ = 0.03). G1: 0/5 (0%), G2a: 6/50 (12%), G2b: 4/22 (18%) and G3: 15/38 (39%) ($P$ = 0.03). None of the G4 tumours were informative for the $(CA)_n$ repeat. The observation that was made for the $(CA)_n$ repeat and LOH was also true for the $(AAAAT)_n$ repeat. Tis: 0/2 (0%), Ta: 10/56 (18%), T1: 8/19 (42%), ≥ T2: 13/26 (50%) ($P$ = 0.01). G1: 0/7 (0%), G2a: 6/40 (15%), G2b: 7/18 (39%), G3: 16/36 (44%) and G4: 2/2 (100%) ($P$ = 0.02). The correlation between LOH and tumours of high stage and grade is shown in Figure 2, panels C–F. Combined stage and grade information shows that 6/51 (12%) of lowly malignant tumours had LOH for the $(CA)_n$ repeat compared to 19/64 (30%) of highly malignant tumours ($P$ = 0.02). 5 out of 43 (12%) lowly malignant tumours had LOH for the $(AAAAT)_n$ repeat compared to 26/60 (43%) of highly malignant tumours ($P$ = 0.0006).

### DISCUSSION

We found a clear distinction between G2a and G2b urinary bladder neoplasms concerning the occurrence of genetic events related to *p53*. Virtually no mutations (exons 5–8) occurred in TaG1–G2a tumours. Overall we discovered 31 p53 mutations in 26 patients (14%) in 189 analysed bladder neoplasms and depending on the used marker, LOH was found in 22% and 30% of informative cases. A substantial part of urinary bladder neoplasms showed no genetic alterations in *p53*. Only one mutation (1.2%) was found in a lowly malignant tumour (Ta, G1–G2a) whereas 25 (24%) of invasive (≥T1) or high grade (G2b–G4) tumours were mutated. This is the first series (to our knowledge) relating the occurrence of a genetic event in p53 to the refined grade categories 2a and 2b. Our data support the notion that moderately differentiated bladder neoplasms are genetically heterogeneous and that it may be beneficial to abandon the present WHO category, as suggested by an international panel of pathologists (Epstein et al, 1998).

The Li–Fraumeni syndrome, associated with an inherited p53 mutation, has a very low rate (in the vicinity of 1%) of urinary bladder neoplasms, which suggests that a p53 mutation is more likely to be a late event in bladder tumour progression than an initiator of malignant transformation (Kleihues et al, 1997; Varley et al, 1997). Other studies of urinary bladder cancer have reported mutation frequencies for p53 from 6% to 61% (Sidransky et al, 1991; Shipman et al, 1997) and most studies have found higher mutation frequencies than we did. Where most published studies have focused on tumours of high stage and grade, our patient series presented many tumours of low stage and grade.

We found more transitions than transversions. The most common mutation was the G:C→A:T transition which can occur both by spontaneous deamination of 5-methylcytosine to thymine and factor mediation, e.g., through oxygen radicals or nitric oxide (Lindahl, 1979). Although urinary bladder cancer has a strong association with cigarette smoke we found only one G:C→T:A transversion, a mutation present at an increased frequency in lung cancers among smokers (IARC, 1985; Bennett et al, 1999). Reported series of urinary bladder cancer shows 14% of G:C→C:G transversions, 12% G:C→T:A transversions and 19% of A:T→G:C transitions; we found 19%, 3% and 10% respectively (Hollstein et al, 1998). We did not find any A:T→C:G transversions, but this mutation has a low overall frequency (3%) and we only found one deletion (3%), which is less than in reported series (10%) (Hollstein et al, 1998). We found 6 mutations at CpG sites (19%), which is lower compared to other cancers (23%) but in agreement with previously reported series for urinary bladder (18%). However, urinary bladder neoplasms caused by endemic schistosomal infection (mainly squamous cell carcinoma) have a higher reported frequency of CpG mutations (Warren et al, 1995; Hainaut and Hollstein, 2000). One sample had 3 mutations in exon 8, this sample also showed LOH for the $(AAAAT)_n$ repeat indicating that all the 3 mutations were in the same allele. All 3 mutations were G:C→A:T transitions located in exon 8 between codons 285 and 291 and probably caused by the same mutational event. 4 mutations (13%) were found in codon 280 and 2 (6.5%) in codon 285, these 2 codons have been previously reported as mutational hot spots for urinary bladder cancer (Spruck et al, 1993; Xu et al, 1997). In the p53 database, where over 10 000 p53 mutations have been reported, the codon 280 mutation makes up for 1.2% of all reported mutations but 5.1% of urinary bladder cancer mutations (Hainaut et al, 1998). Moreover, gallbladder has 8% of codon 280 mutations and neoplasms in the urinary system 2.7%. No other cancer, where at least 50 mutations have been reported totally, has a frequency over 2% for codon 280 mutations. Mutations in codon 285 represent 0.8% of mutations in all cancers but 4.3% of urinary bladder cancer mutations.

31 samples (21%) informative for one or both markers showed LOH for either the $(AAAAT)_n$ repeat or the $(CA)_n$ repeat but were not mutated in any of the exons 5–8. Although SSCP does not guarantee 100% mutation detection, the SSCP method we used has been previously evaluated in 4 different gels screening for known mutations and had a sensitivity of over 90% (Berggren et al, 2000b). Following Knudson's 'two-hit' hypothesis (Knudson, 1971), one could expect a mutation in exons that were not analysed in this study or mutations in distant splice sites and promoter regions. This seems unlikely considering that the mutation frequency for exons 2–4 and 9–11 is much lower than for exons 5–8 (Miyamoto et al, 1993; Lianes et al, 1994; Williamson et al, 1994; Hainaut and Hollstein, 2000) and that 31 samples had LOH but no mutation. Another explanation could be a second tumour suppressor gene on chromosome 17p13 such as the suggested tumour suppressor gene *HIC-1* (Wales et al, 1995). LOH has also been reported at 17p13.3 for various types of cancer including breast and ovarian cancer (Coles et al, 1990; Schultz et al, 1996). It is also possible that the allelic loss at chromosome 17p13.1 may precede the *p53* gene mutation as suggested by others for chronic myelogenous leukaemias (Feinstein et al, 1991; Nakai et al, 1992). However, since the $(AAAAT)_n$ repeat is intragenic to *p53*, it is unlikely that this marker would reflect another tumour suppressor gene and the most possible explanation for our findings is that other mechanisms than point mutations inactivate p53. It is likely to believe that genes upstream or downstream of *p53* have been inactivated, such as *MDM2* and $p14^{ARF}$, thereby disrupting the *p53* pathway. Moreover, loss or inactivation of the wild-type allele is not always required for disruption of *p53* function; a number of mutants can inactivate wild-type *p53* in a dominant manner so that the mutant *p53* can oligomerize with wild-type *p53* to form an inactive complex (Hainaut and Hollstein, 2000).

Although only 14% of the analysed samples carried mutations, 30% showed LOH for the intragenic $(AAAAT)_n$ repeat suggesting that *p53* is affected in urinary bladder cancer also through mechanisms other than mutations. To understand the pathogenesis and cancer aetiology of urinary bladder cancer, further studies of other key components in the cell cycle machinery, for example the genes *p16*, $p14^{ARF}$ and *RB* are required in large population based materials. Our data support that grade 2a tumours, recently suggested to be renamed as grade 1, seldom harbour a *p53*

mutation and may have occurred through different genetic events than grade 2b–4 urinary bladder neoplasms – which in many cases harbour a *p53* mutation, allele loss and possibly some other genetic event that results in a dysfunctional p53 protein.

### ACKNOWLEDGEMENTS

The authors wish to thank Dr Rajiv Kumar for helpful discussions and Dr Guogang Xu for help with statistical calculations. This study was supported by a grant from the Swedish Cancer Society and the Stockholm Cancer Foundation.

### REFERENCES

Bennett WP, Hussain SP, Vahakangas KH, Khan MA, Shields PG and Harris CC (1999) Molecular epidemiology of human cancer risk: gene-environment interactions and p53 mutation spectrum in human lung cancer. *J Pathol* **187**: 8–18

Berggren P, Hemminki K and Steineck G (2000a) p53 intron 7 polymorphisms in urinary bladder cancer patients and controls. *Mutagenesis* **15**: 57–60

Berggren P, Steineck G and Hemminki K (2000b) A rapid fluorescence based multiplex polymerase chain reaction – single-strand conformation polymorphism method for p53 mutation detection. *Electrophoresis* **21**: 2335–2342

Bergkvist A, Ljungqvist A and Moberger G (1965) Classification of bladder tumours based on the cellular pattern. Preliminary report of a clinical-pathological study of 300 cases with a minimum follow-up of eight years. *Acta Chir Scand* **130**: 371–378

Bringuier PP, McCredie M, Sauter G, Bilous M, Stewart J, Mihatsch MJ, Kleihues P and Ohgaki H (1998) Carcinomas of the renal pelvis associated with smoking and phenacetin abuse: p53 mutations and polymorphism of carcinogen-metabolising enzymes. *Int J Cancer* **79**: 531–536

Carbin BE, Ekman P, Gustafson H, Christensen NJ, Sandstedt B and Silfversward C (1991) Grading of human urothelial carcinoma based on nuclear atypia and mitotic frequency. I. Histological description. *J Urol* **145**: 968–971

Carbone D, Chiba I and Mitsudomi T (1991) Polymorphism at codon 213 within the p53 gene. *Oncogene* **6**: 1691–1692

Centre for epidemiology (1998) *Cancer incidence in Swede 1996* The national board of health and welfare: Stockholm

Cohen SM, Samuel MC, Tomoyuki S and Steinech G (2000) Epidemiology and etiology of premalignant and malignant urothelial changes. *Scand J Urol Nephrol* **205**: 105–115

Coles C, Thompson AM, Elder PA, Cohen BB, Mackenzie IM, Cranston G, Chetty U, Mackay J, Macdonald M, Nakamura Y, Hoyhtim B and Steel CM (1990) Evidence implicating at least two genes on chromosome 17p in breast carcinogenesis. *Lancet* **336**: 761–763

Epstein JI, Amin MB, Reuter VR and Mostofi FK (1998) The World Health Organization/International Society of Urological Pathology consensus classification of urothelial (transitional cell) neoplasms of the urinary bladder. Bladder Consensus Conference Committee. *Am J Surg Pathol* **22**: 1435–1448

Esposti PL and Zajicek J (1972) Grading of transitional cell neoplasms of the urinary bladder from smears of bladder washings. A critical review of 326 tumors. *Acta Cytol* **16**: 529–537

Feinstein E, Cimino G, Gale RP, Alimena G, Berthier R, Kishi K, Goldman J, Zaccaria A, Berrebi A and Canaani E (1991) p53 in chronic myelogenous leukemia in acute phase. *Proc Natl Acad Sci USA* **88**: 6293–6297

Fields, S and Jang SK (1990) Presence of a potent transcription activating sequence in the p53 protein. *Science* **249**: 1046–1049

Fujimoto K, Yamada Y, Okajima E, Kakizoe T, Sasaki H, Sugimura T and Terada M (1992) Frequent association of p53 gene mutation in invasive bladder cancer. *Cancer Res* **52**: 1393–1398

Futreal PA, Barrett JC and Wiseman RW (1991) An Alu polymorphism intragenic to the TP53 gene. *Nucleic Acids Res* **19**: 6977

Greenblatt MS, Bennett WP, Hollstein M and Harris CC (1994) Mutations in the p53 tumor suppressor gene: clues to cancer etiology and molecular pathogenesis. *Cancer Res* **54**: 4855–4878

Hainaut P and Hollstein M (2000) p53 and human cancer: the first ten thousand mutations. *Adv Cancer Res* **77**: 81–137

Hainaut P, Hernandez T, Robinson A, Rodriguez-Tome P, Flores T, Hollstein M, Harris CC and Montesano R (1998) IARC Database of p53 gene mutations in human tumors and cell lines: updated compilation, revised formats and new visualisation tools. *Nucleic Acids Res* **26**: 205–213

Hall RR and Prout GR (1990) Staging of bladder cancer: is the tumor, node, metastasis system adequate? *Semin Oncol* **17**: 517–523

Hollstein M, Moeckel G, Hergenhahn M, Spiegelhalder B, Keil M, Werle-Schneider G, Bartsch H and Brickmann J (1998) On the origins of tumor mutations in cancer genes: insights from the p53 gene. *Mutat Res* **405**: 145–154

IARC (1985) IARC Monographs on the Evaluation of the Carcinogenic Risk of Chemicals to Humans, Vol. 38. pp. 244–270

Isobe M, Emanuel BS, Givol D, Oren M and Croce CM (1986) Localization of gene for human p53 tumour antigen to band 17p13. *Nature* **320**: 84–85

Jones MH and Nakamura Y (1992) Detection of loss of heterozygosity at the human TP53 locus using a dinucleotide repeat polymorphism. *Genes Chromosomes Cancer* **5**: 89–90

Kern SE, Kinzler KW, Bruskin A, Jarosz D, Friedman P, Prives C and Vogelstein B (1991) Identification of p53 as a sequence-specific DNA-binding protein. *Science* **252**: 1708–1711

Kleihues P, Schauble B, zur Hausen A, Esteve J and Ohgaki H (1997) Tumors associated with p53 germline mutations: a synopsis of 91 families. *Am J Pathol* **150**: 1–13

Knowles MA (1999a) The genetics of transitional cell carcinoma: progress and potential clinical application. *BJU Int* **84**: 412–427

Knowles MA (1999b) Identification of novel bladder tumour suppressor genes. *Electrophoresis* **20**: 269–279

Knudson AG Jr (1971) Mutation and cancer: statistical study of retinoblastoma. *Proc Natl Acad Sci USA* **68**: 820–823

Lane DP and Crawford LV (1979) T antigen is bound to a host protein in SV40-transformed cells. *Nature* **278**: 261–263

Lianes P, Orlow I, Zhang ZF, Oliva MR, Sarkis AS, Reuter VE and Cordon-Cardo C (1994) Altered patterns of MDM2 and TP53 expression in human bladder cancer [see comments]. *J Natl Cancer Inst* **86**: 1325–1330

Lindahl T (1979) DNA glycosylases, endonucleases for apurinic/apyrimidinic sites, and base excision-repair. *Prog Nucleic Acid Res Mol Biol* **22**: 135–192

Lohmann D, Putz B, Reich U, Bohm J, Prauer H and Hofler H (1993) Mutational spectrum of the p53 gene in human small-cell lung cancer and relationship to clinicopathological data. *Am J Pathol* **142**: 907–915

Malmstrom PU (1988) Prognosis of transitional cell bladder carcinoma. With special reference to ABH blood group isoantigen expression and DNA analysis. *Scand J Urol Nephrol Suppl* **112**: 1–55

Malmstrom PU, Busch C and Norlen BJ (1987) Recurrence, progression and survival in bladder cancer. A retrospective analysis of 232 patients with greater than or equal to 5-year follow-up. *Scand J Urol Nephrol* **21**: 185–195

Miyamoto H, Kubota Y, Shuin T, Torigoe S, Hosaka M, Iwasaki Y, Danenberg K and Danenberg PV (1993) Analyses of p53 gene mutations in primary human bladder cancer. *Oncol Res* **5**: 245–249

Nakai H, Misawa S, Toguchida J, Yandell DW and Ishizaki K (1992) Frequent p53 gene mutations in blast crisis of chronic myelogenous leukemia, especially in myeloid crisis harboring loss of a chromosome 17p. *Cancer Res* **52**: 6588–6593

Prosser J and Condie A (1991) Biallelic ApaI polymorphism of the human p53 gene (TP53). *Nucleic Acids Res* **19**: 4799

Raycroft L, Wu HY and Lozano G (1990) Transcriptional activation by wild-type but not transforming mutants of the p53 anti-oncogene. *Science* **249**: 1049–1051

Sambrook J, Fritschookstein EF and Maniatis T (1989) *Molecular cloning. A laboratory manual*. Cold Spring Harbour Press.

Schultz DC, Vanderveer L, Berman DB, Hamilton TC, Wong AJ and Godwin AK (1996) Identification of two candidate tumor suppressor genes on chromosome 17p13.3 *Cancer Res* **56**: 1997–2002

Shipman R, Schraml P, Moch H, Colombi M, Sauter G, Mihatsch M and Ludwig C (1997) p53 protein accumulation and p53 gene alterations (RFLP, VNTR and p53 gene mutations) in non-invasive versus invasive human transitional bladder cancer. *International Journal of Oncology* **10**: 801–806

Sidransky D, Von Eschenbach A, Tsai YC, Jones P, Summerhayes I, Marshall F, Paul M, Green P, Hamilton SR, Frost P et al (1991) Identification of p53 gene mutations in bladder cancers and urine samples. *Science* **252**: 706–709

Spruck CHd, Rideout WMd, Olumi AF, Ohneseit PF, Yang AS, Tsai YC, Nichols PW, Horn T, Hermann GG, Steven K, Ross RK, Yu MC and Jones PA (1993) Distinct pattern of p53 mutations in bladder cancer: relationship to tobacco usage [published erratum appears in Cancer Res 1993 May 15;53 (10 Suppl): 2427]. *Cancer Res* **53**: 1162–1166

Steineck G, Wiholm BE and Gerhardsson de Verdier M (1995) Acetaminophen, some other drugs, some diseases and the risk of transitional cell carcinoma. A population-based case-control study. *Acta Oncol* **34**: 741–748

Wada T, Louhelainen J, Hemminki K, Adolfsson J, Wijkstrom H, Norming U, Borgstrom E, Hansson J, Sandstedt B and Steineck G (2000) Bladder cancer:

 *© 2001Cancer Research Campaign*

allelic deletions at and around the retinoblastoma tumor suppressor gene in relation to stage and grade. *Clin Cancer Res* **6**: 610–615

Wales MM, Biel MA, el Deiry W, Nelkin BD, Issa JP, Cavenee WK, Kuerbitz SJ and Baylin SB (1995) p53 activates expression of HIC-1, a new candidate tumour suppressor gene on 17p13.3. *Nat Med* **1**: 570–577

Varley JM, Evans DG and Birch JM (1997) Li-Fraumeni syndrome-a molecular and clinical review. *Br J Cancer* **76**: 1–14

Warren W, Biggs PJ, el-Baz M, Ghoneim MA, Stratton MR and Venitt S (1995) Mutations in the p53 gene in schistosomal bladder cancer: a study of 92 tumours from Egyptian patients and a comparison between mutational spectra from schistosomal and non-schistosomal urothelial tumours. *Carcinogenesis* **16**: 1181–1189

Williamson MP, Elder PA and Knowles MA (1994) The spectrum of TP53 mutations in bladder carcinoma. *Genes Chromosomes Cancer* **9**: 108–118

Xu X, Stower MJ, Reid IN, Garner RC and Burns PA (1997) A hot spot for p53 mutation in transitional cell carcinoma of the bladder: clues to the etiology of bladder cancer. *Cancer Epidemiol Biomarkers Prev* **6**: 611–616

*© 2001 Cancer Research Campaign*
